# Estimating power corrections for the Drell–Yan process

**DOI:** 10.1140/epjs/s11734-025-02073-1

**Published:** 2025-12-10

**Authors:** Ekta Chaubey, Pooja Mukherjee

**Affiliations:** 1 Bethe Center for Theoretical Physics, Universität Bonn, 53115 Bonn, Germany; 2 Institut für Theoretische Physik, Universität Hamburg, Luruper Chaussee 149, 22761 Hamburg, Germany

## Abstract

We study power corrections in the Drell–Yan (DY) process using state-of-the-art predictions for both neutral and charged current production. For both types of DY processes, we account for power corrections arising from bottom and charm quark effects within a variable flavor number scheme. Our results show that these corrections become significant in the low-*Q* region. We also ensure proper treatment of overlapping contributions by carefully applying matching procedures to eliminate any double counting.

## Introduction

High-energy hadron–hadron colliders offer unique opportunities to probe fundamental physics, from searches for beyond-the-Standard Model particles to precision studies of known Standard Model (SM) processes. A key benchmark process in this context is the Drell–Yan (DY) process, which facilitates lepton pair production via neutral-current (NC) and charged-current (CC) interactions. DY measurements across a wide range of collider energies have provided critical insights into hadron structure, SM parameters, and the validity of factorization theorems. The LHC, with its unprecedented precision, has further refined our understanding, and its upcoming high-luminosity phase promises even greater improvements. These experimental advancements, in turn, drive the need for increasingly precise theoretical predictions. This need translates to a complete understanding of the DY process for the future precision era. For a comprehensive review of the state-of-the-art QCD corrections to this process, see [1]. Of course, QED corrections also play a crucial role, as they significantly affect the determination of the lepton-pair transverse momentum spectrum and compete with quark mass effects; see, for example, [2, 3]. A related phenomenological analysis focusing on an improved description of the bottom quark is presented in Ref. [4].

Recent progress includes the calculation of the inclusive DY cross-section up to $\text {N}^3\text {LO}$ in perturbative QCD, as well as differential predictions incorporating electroweak(EW) and mixed QCD-EW corrections, quark mass effects, and low-transverse momentum dynamics. Parton distribution function (PDF) extractions have also reached new levels of accuracy, with partial $\text {N}^3\text {LO}$ results available. A particularly interesting, but unexplored kinematic regime at the LHC is the low invariant mass and forward rapidity region, accessible via LHCb. This region is sensitive to small *x* (parton momenta fractions) of PDFs at low virtuality but also presents theoretical challenges due to slower perturbative convergence of partonic cross-sections and potential quark mass effects.

In this contribution, we estimate the power corrections to the inclusive NCDY and CCDY cross-sections using state-of-the-art theoretical predictions. We also outline the method used to extract these power corrections within the massive variable flavor number scheme (MVFNS). This contribution serves as a prelude to [1], where a comprehensive analysis is presented. In particular, the sources of uncertainty in DY are examined in detail for both NC and CC processes, with particular emphasis on the low invariant mass region. Differential predictions for the invariant mass spectrum are provided at N$^3$LO, supplemented by exact charm and bottom quark mass effects at $\mathcal {O}(\alpha _s^{2})$. Further investigations on the impact of PDF choices (including approximate $\hbox {N}^{3}\hbox {LO}$ sets), scale variations, the strong coupling constant, and heavy-quark mass effects on the resulting distributions is also carried out.

The writeup is structured as follows: Sect. 2 introduces the computational framework for both NC and CC DY processes, detailing the theoretical fixed-order calculations and the matching procedure employed to combine massive and massless calculations. Section 3 presents the results of the MVFNS corrections, before the conclusions in Sect. 4.

## Theoretical setup

We start with the theoretical set up of the DY process. Let there be two protons (P) scattering with momenta $P_1$ and $P_2$, and let *Q* be the invariant mass of the final state particles $L_1 L_2$. Then the process is given as:

1 $$\begin{aligned} \mathrm{{P}}(P_1) + \mathrm{{P}} (P_2) \rightarrow L_1 L_2 (Q)+ X. \end{aligned}$$

where $L_1L_2$ are lepton pairs ($l^{+}l^{-}$) for NCDY and lepton–­neutrino pairs ($l^{\pm }\nu _l$) for CDY. Here, *X* denotes the additional QCD radiation. The cross-section is mediated through a virtual *Z* boson or a photon in case of NCDY and a virtual *W*-boson for CCDY to decay subsequently into a pair of lepton (lepton–neutrino for CCDY) for NCDY in the final state.

Let $S= {(P_1+P_2)}^2$ be the incoming energy of the protons. The hadronic differential cross-section for DY production with respect to *Q* can be written using the QCD factorization theorem as

2 $$\begin{aligned} Q^2 \; \frac{d \sigma ^{(n_f, \kappa )}}{dQ^2}= \tau \sum _{i,j} \mathcal {L}_{ij}^{(n_f)} (\tau , \mu _F) \otimes \eta _{ij}^{(n_f,\kappa )}(\tau , a_s^{(n_f)}(\mu _R)). \end{aligned}$$

Here $\tau$ is the hadronic scaling variable, defined as $\tau = \frac{Q^2}{S}$ and $\kappa \in \{\pm \,1,0\}$, depending on whether the process is mediated by a neutral vector boson ($\kappa =0$) or a $W^{\pm }$ ($\kappa =\pm 1$). $\mu _F$ and $\mu _R$ denote the factorization scale and the renormalization scale, respectively. The $\eta _{ij}^{(n_f,\kappa )}$ denotes the partonic cross section for producing a lepton pair or a lepton–neutrino pair from a collision of two partons *i* and *j*. The sum runs over all active partons in the proton, i.e., gluons and all massless quark flavors. The partonic luminosity $\mathcal {L}_{ij}^{(n_f)}$ is the convolution over the $n_f$-flavor PDFs:

3 $$\begin{aligned} \mathcal {L}_{ij}^{(n_f)} (\tau , \mu _F)&= f_i^{(n_f)} (\tau , \mu _F) \otimes f_j^{(n_f)} (\tau ,\mu _F) \nonumber \\ &\equiv \int _0^1 f_i^{(n_f)} (x_1, \mu _F)\; f_j^{(n_f)} (x_2, \mu _F)\; \delta (\tau - x_1 x_2)\; \textrm{d}x_1 \textrm{d}x_2. \end{aligned}$$

The $f_i^{(n_f)}(x_i, \mu _F)$ represents the PDFs, i.e., the probability of finding a parton *i* with momentum fraction $x_i$ inside the proton. The partonic cross-section $\eta _{ij}^{(n_f, \kappa )}$ exhibits a perturbative expansion in the strong coupling $a_s$, with $a_s= \frac{\alpha _s}{\pi }$, given by

4 $$\begin{aligned} \eta _{ij}^{(n_f,\kappa )} (\tau , a_s^{(n_f)}(\mu _R)) = \sum _{k = 0}^\infty a_s^{(n_f) k} (\mu _R) \;\eta _{ij}^{(n_f, \kappa , k)} (\tau ). \end{aligned}$$

### The matching procedure for Drell–Yan process

In this section, we discuss the matching procedure for DY process. We start by discussing the NCDY process. In matrix element computations involving bottom quarks, they can be treated as either massless or massive. The 5-flavor scheme (5FS) assumes all quarks are massless, including the bottom quark, which has an associated PDF and contributes to QCD evolution with $n_f=5$. However, neglecting the bottom mass in final-state processes involving massive particles like the Z boson introduces infrared and collinear singularities, as mass effects are resummed inside the PDFs in the 5FS cross-section.

Alternatively, the 4-flavor scheme (4FS) treats the bottom quark as massive and the 3-flavor scheme (3FS) treats both bottom and charm quarks massive. This means that they appear only in the final states and have no PDFs. In these schemes, both bottom and charm quarks arise from gluon splittings at the leading order and all finite-mass effects are included in the partonic cross section. While this avoids collinear divergences, it introduces large logarithms of the form $\log \frac{Q^2}{m_Q^2}$ which can spoil perturbative convergence, where $m_Q \in \{m_c,m_b\}$. These logarithms are resummed in the 5FS, improving predictive accuracy. Representative diagrams contributing to various orders in perturbation theory for 4FS is shown in Table 1.

**Table 1:**
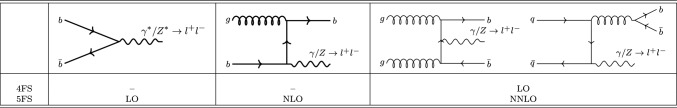
Representative diagrams for the NCDY process at different orders in perturbation theory in the 4FS and 5FS

*AE* adverse event, *BCPNN* Bayesian Confidence Propagation Neural Network, *MCSG* Markov Chain Signal Generation *PRR* proportional reporting ratios, *ROR* reporting odds ratio

The results in the massless limit are known up to $\text {N}^3\text {LO}$ [5], whereas the massive ones have been computed up to NNLO in Ref. [6]. The matched calculation for Higgs production in bottom quark annihilation was performed using the FONLL scheme [7] up to $\text {N}^3\text {LO}$ in Ref. [8]. Studies on *Z* boson production in bottom quark fusion using the same FONLL procedure were performed in Ref. [9]. The operator-matrix elements (OMEs) used in this article were computed in Ref. [10]. The matching procedure adopted in our article was first performed in Ref. [11] for NCDY.

#### Estimating the mass corrections in DY

In the MVFNS, we combine massless DY with the power correction terms extracted from the massive computations, as outlined below. For brevity, from now on we denote $a_s^{(n_f) k}$ as $a_s$. The cross-section in the MVFNS is defined as:

5 $$\begin{aligned} \mathrm{{d}} \sigma ^\mathrm{{MVFNS}} = \mathrm{{d}} \sigma ^{(5,\kappa )} + \sum _{i = c,b}^{n_f} \mathrm{{d}} \sigma _{i,pc}^{(5,\kappa )}. \end{aligned}$$

This construction of d$\sigma ^\mathrm{{MVFNS}}$ includes the resummation of massive collinear logarithms to all orders in $a_s$. Additionally, the exact heavy quark mass dependence is incorporated up to the fixed-order accuracy to which the power correction terms $\mathrm{{d}}\sigma _{i,pc}^{(5,\kappa )}$ is computed. The first term on the right-hand side, $\mathrm{{d}} \sigma ^{(5,\kappa )}$, is obtained from massless computations using n3loxs, while the second term is derived using the procedure detailed below.

To provide a uniform definition of NCDY scattering across all energy scales, we combine the 3FS, 4FS and 5FS schemes. As an example for combining 4FS and 5FS cross sections for NCDY, we extract the power-suppressed terms from Eq. 5 as follows. The massive differential cross-section is expressed as a sum of three components:

6 $$\begin{aligned} \mathrm{{d}}\sigma ^{(4,0)} = \mathrm{{d}}\sigma _{n_f}^{(5,0)} + \mathrm{{d}}\sigma _{\ln [m]}^{(5,0)} + \mathrm{{d}}\sigma _{pc}^{(4,0)}. \end{aligned}$$

Here, $\mathrm{{d}}\sigma ^{(4,0)}$ represents the fully massive contribution from a single heavy-flavor of mass $m_{Q}$, where *Q* could denote either a charm or bottom quark. The components on the right-hand side are:

- $n_f$ **corrections:** $\mathrm{{d}}\sigma _{n_f}^{(5,0)}$ are the $n_f$ contributions arising from a single flavor in the massless limit. In the massless computation, the heavy quark *Q* contributes to the same subprocesses as in the massive computation, but with $m=0$. Thus, these corrections can be directly extracted from the massless partonic cross-section at any given order in $\alpha _s$. We obtained these corrections from the publicly available n3loxs [12]. These terms diverge in the limit $m \rightarrow 0$.
- **Logarithmic corrections:** $\mathrm{{d}}\sigma _{\ln [m]}^{(5,0)}$ are the contributions from massive collinear logarithms. These can be obtained using the decoupling relations for the PDFs and $a_s$. The logarithmic dependence of the massive corrections originates from collinear divergences. These corrections are constructed using only massless inputs by expressing both results in terms of a common set of PDFs and $a_s$ in the 5FS scheme.
- **Power corrections:** $\mathrm{{d}}\sigma _{pc}^{(5,0)}$ are contributions that vanish smoothly in the massless limit. These arise exclusively in the massive computations.

We briefly explain the decoupling relations used to describe parameters such as $\alpha _s$ and PDFs in a theory with a massive quark (4FS) relative to an effective theory where the corresponding quark is treated as massless (5FS) at a fixed order. The strong coupling constant relation between the 4FS and 5FS schemes, matched at scale $\mu _R$, is as given in Ref. [13]. The PDF relation is provided in terms of OMEs ($A_{ij}$), which describe transitions between partonic states $i \rightarrow j$:

7 $$\begin{aligned} f_i^{(5)} = \sum _{j=-4}^4 K_{ij}\big (L_Q, a_s^{(4)}\big ) \otimes f_j^{(4)}, \quad -\,5 \le i \le 5, \end{aligned}$$

where $K_{ij}$ are kernels from [10]. Substituting this relation into above removes the *b*-quark PDF from the initial states and gives rise to contributions to $\mathrm{{d}}\sigma _{\ln [m]}^{(5,0)}$. The contribution up to $O(a_s^2)$ reads as:

8 $$\begin{aligned} \mathrm{{d}}\sigma _{\ln [m]}^{(5,0)} = \tau \sum _{i,j=-4}^4 \mathcal {L}_{ij}^{(5)}(\tau , \mu _F) \otimes \textbf{A}_{ij}(\tau , \mu _F, \mu _R, L_Q, a_s, m), \end{aligned}$$

where $\textbf{A}_{ij} = \eta _{ij}^{(5,0,k)} + \delta \eta _{ij}^{(5,0,k)}$. The expansion in $a_s^k$ for $\delta \eta _{ij}^{(n_f,0,k)}$ is:

9 $$\begin{aligned} \delta \eta _{gg}^{(5,0,2)}&= 2 A_{bg}^{(1)} \otimes A_{bg}^{(1)} \otimes \eta _{q\bar{q}}^{(5,0,0)} + 4 A_{bg}^{(1)} \otimes \eta _{qg}^{(5,0,1)}. \end{aligned}$$

Similar generalizations to 5FS vs 3FS matching terms for NCDY are as follows:

10 $$\begin{aligned} \delta \eta ^{(5,0,2)} _{gg} =&\sum _{i\in \{c,b \}} 2 A_{ig }^{(1)} \otimes A_{ig}^{(1)} \otimes \eta _{q \bar{q}}^{(5,0,0)} + 4 A_{ig}^{(1)} \otimes \eta _{q g}^{(5,0,1)}. \end{aligned}$$

For CCDY ($W^-$ production), these equations attain the form

11 $$\begin{aligned}&\delta \eta _{g{\bar{u}}}^{(5,-1, 1)} = A_{bg}^{(1)}\otimes \eta _{b{\bar{u}}}^{(5,-1,0)},\nonumber \\ &\delta \eta _{gi}^{(5,-1,1)} = A_{cg}^{(1)}\otimes \eta _{{\bar{c}} i}^{(5,-1,0)} , \quad i \in \{ d,s\} \end{aligned}$$

Here the one-loop OME is given as,

12 $$\begin{aligned} A_{Qg}^{(1)}&= -2 \bigg ( z^2 + (1-z)^2 \bigg )\ln {\frac{m_Q^2}{\mu ^2}}, \quad Q \in \{ c,b\} \end{aligned}$$

All these convolutions are computed analytically using PolyLogTools [14], expressed in terms hyper-logarithmic (HPL) functions which are later on computed numerically using the computer algebra system GiNaC [15]. The massive corrections are computed using in-house code for NCDY and MCFM-10.3 [16, 17] for CCDY. For further clarifications on estimating the mass corrections in DY we refer the interested readers to [1].

## Results

In this section, we estimate the impact of power correction terms derived from 4FS and 3FS massive corrections. To present the phenomenological results for the binned invariant-mass distribution we use the following notation. Each term in Eq. (6) can be integrated between two scales $Q_{\text {min}}$ and $Q_{\text {max}}$, which we denote using the notation $\Sigma ^{(n_f,\kappa )}$ , which is schematically given as

13 $$\begin{aligned} \Sigma ^{(n_f,\kappa )}(Q_{\text {min}},Q_{\text {max}}) = \int _{Q_{\text {min}}^{2}}^{Q_{\text {max}}^{2}}{{\textrm{d Q}}^{2}}\,\frac{\textrm{d}\mathrm{\sigma }^{(n_f,\kappa )}}{{\textrm{dQ}}^{2}}\,. \end{aligned}$$

Here we suppress the dependence of all quantities on the renormalization and factorization scales. Expanding the above quantity in terms of $a_s$ we write:

14 $$\begin{aligned} \Sigma ^{(n_f,\kappa )}_{{\textrm{N}}^{k}{\textrm{LO}}}(Q_{\textrm{min}},Q_{\textrm{max}}) = \sum _{l=0}^k a_s^{(n_f)}(\mu _R)^l\,\Sigma ^{(n_f,\kappa ,l)}(Q_{\textrm{min}},Q_{\textrm{max}})\,. \end{aligned}$$

After verifying the matching procedure between different flavor schemes, as established in Sect. 2.1, we assess the effects of power corrections arising from the massive corrections.

**Fig. 1:**
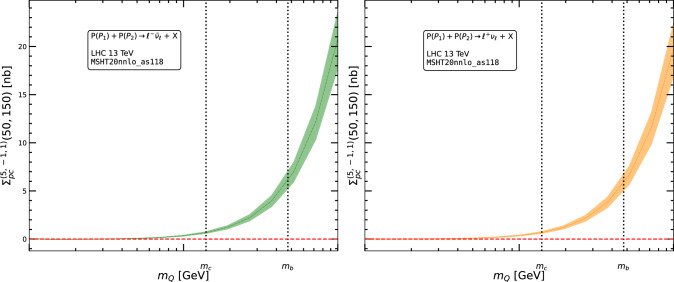
Power-corrections to the CCDY process up to NLO accuracy for the *Q*-bin (50 GeV, 150 GeV). The bands represent the 7-point variation of $\mu _R$ and $\mu _F$ around the central (dynamic) scale $\mu _R=\mu _F=Q$. We observe that the power corrections vanish in the limit $m_Q \rightarrow 0$

**Fig. 2:**
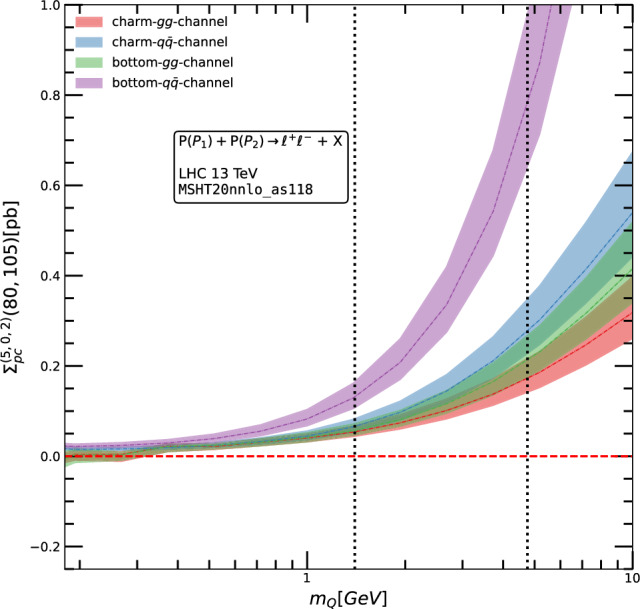
Massive power-corrections to the NCDY cross-section in the $Q \in [80, 105]$ GeV. The bands represent the 7-point variation of $\mu _R$ and $\mu _F$ around the central (dynamic) scale $\mu _R=\mu _F=Q$. We observe that the power corrections vanish in the limit $m_Q \rightarrow 0$

For example, in Figs. 1 and 2, we present the power corrections for CCDY at NLO the *gg* and $q\bar{q}$ channels: at NNLO for NCDY. The plots demonstrate that the power correction terms vanish in the limit $m_Q \rightarrow 0$, confirming the expected behavior. In addition to validating the matching procedure, we also display the scale variation of the power correction terms separately for the *gg* and $q\bar{q}$ channels. This is shown within the LHCb fiducial region, for $Q \in [80, 105 ]$ GeV in Fig. 2, and for $Q \in [50, 150 ]$ GeV in Fig. 1.

**Fig. 3:**
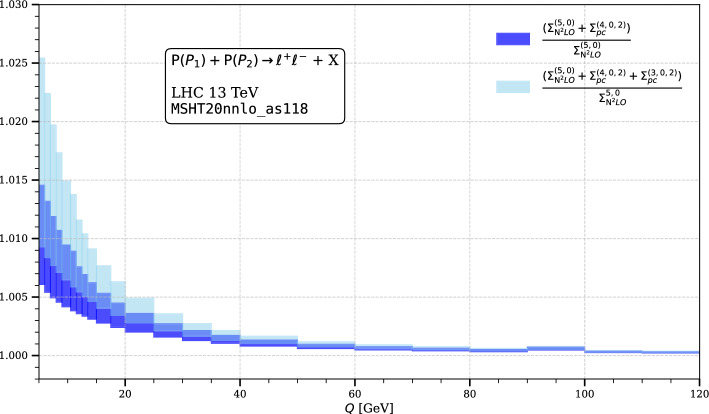
The plot shows the impact of the power corrections on the invariant-mass distribution of the NCDY process. The cross sections are obtained with MSHTnnlo_as118 PDF set

We also plot the impact of the extracted power correction terms over the entire fiducial region $Q \in [4,120]$ GeV in Fig. 3, comparing them with respect to the NNLO NCDY cross-section.

We observe that the 3FS contributes more than the 4FS. This implies that the combined mass effects of charm and bottom has more effects on the cross section, as compared with only bottom mass effects. This is particularly true for the low-*Q* region. We also provide a numerical estimate of the power corrections for physical quark mass values, using $m_b =4.75$ GeV and $m_c = 1.4$ GeV, as shown in Table 2.

**Table 2:** Predictions from the 5FS and the massive power-corrections at are shown for the central scale $\mu _R=\mu _F=Q$ for physical values of quark masses

Process	$\Sigma ^{(n_f,0)}(Q_{\text {min}},Q_{\text {max}})$	Prediction (pb)
NCDY	$\Sigma _{\text {N}^2\text {LO}}^{(5,0)}(80,105)$	1824.63
	$\Sigma _{pc}^{(4,0,2)}(80,105)$	0.965396
	$\Sigma _{pc}^{(3,0,2)}(80,105)$	0.088011
CCDY	$\Sigma _{\text {N}\text {LO}}^{(5,+1)}(50,150)$	11482.76
	$\Sigma _{pc}^{(3,+1,1)}(50,150)$	0.662841
CCDY	$\Sigma _{\text {N}\text {LO}}^{(5,-1)}(50,150)$	8524.48
	$\Sigma _{pc}^{(3,-1,1)}(50,150)$	0.662637

## Conclusion

In this contribution, we analyze the impact of power correction terms arising from bottom and charm quark masses at NNLO for the NCDY process and at NLO for CCDY. In the low-*Q* region, the corrections for NCDY can be as large as 2.5–5% within the range $Q \in [4,30]$ GeV. For CCDY, the power corrections are much smaller—around 0.006% in the range $Q \in [50,150]$ GeV. In the process of determining these corrections, we also verify the consistency of the matching scheme presented here.

## Data Availability

No data were associated in the manuscript.
